# Weight-Bearing CT: Advancing the Diagnosis and Treatment of Hallux Valgus, Midfoot Pathology, and Progressive Collapsing Foot Deformity

**DOI:** 10.3390/diagnostics15030343

**Published:** 2025-01-31

**Authors:** Dong-Il Chun, Jaeho Cho, Sung Hun Won, Otgonsaikhan Nomkhondorj, Jahyung Kim, Chi Young An, Young Yi

**Affiliations:** 1 Department of Orthopaedic Surgery, Soonchunhyang University Seoul Hospital, 59, Daesagwan-ro, Yongsan-gu, Seoul 04401, Republic of Korea; orthochun@gmail.com (D.-I.C.); orthowon@gmail.com (S.H.W.); 132523@schmc.ac.kr (C.Y.A.); 2 Department of Orthopaedic Surgery, Chuncheon Sacred Heart Hospital, Hallym University, 77, Sakju-ro, Chuncheon-si 24253, Republic of Korea; hohotoy@nate.com; 3 Institute for Skeletal Aging and Orthopedic Surgery, Chuncheon Sacred Heart Hospital, Hallym University, 77, Sakju-ro, Chuncheon-si 24253, Republic of Korea; otgonsaikhan0899@gmail.com; 4 Department of Orthopaedic Surgery, Seoul National University Hospital, Seoul National University College of Medicine, Seoul 03080, Republic of Korea; hpsyndrome@naver.com; 5 Department of Orthopaedic Surgery, Sanggye Paik Hospital, Inje University College of Medicine, Seoul 01757, Republic of Korea; 6 Department of Orthopaedic Surgery and Rehabilitation, Yale School of Medicine, New Haven, CT 06510, USA

**Keywords:** weight bearing, CT, hallux valgus, lisfranc injury, midfoot osteoarthritis, progressive collapsing foot deformity

## Abstract

Since its introduction, weight-bearing computed tomography (WBCT) has gained prominence due to its ability to produce accurate three-dimensional images under natural loading conditions, making it particularly useful for assessing complex foot deformities. This review aimed to focus on the diseases of the foot and categorized the pathological conditions into forefoot disease (hallux valgus), midfoot disease (Lisfranc injuries and midfoot osteoarthritis), and progressive collapsing foot deformity. For each category, the authors detail how WBCT enhances diagnostic accuracy and informs treatment strategies. In hallux valgus, WBCT allows for more precise measurement of established parameters and reveals crucial information about metatarsal pronation and ray instability. For midfoot pathologies, WBCT’s superiority in detecting subtle Lisfranc injuries and characterizing midfoot osteoarthritis is emphasized, highlighting the development of novel measurement techniques. The review extensively covers the application of WBCT in assessing the complex three-dimensional features of PCFD, including hindfoot valgus, midfoot/forefoot abduction, medial column instability, peritalar subluxation, and valgus tilting, presenting several WBCT-specific measurements and the use of distance mapping to quantify joint surface interaction. The authors conclude that WBCT, potentially enhanced through integration with artificial intelligence (AI), represents a significant advancement in foot and ankle care, promising improved diagnostic accuracy, streamlined treatment planning, and, ultimately, better patient outcomes.

## 1. Introduction

Over the past decade, accurate assessment of complex foot deformities remains challenging, often hampered by the limitations of conventional radiography, which provides only two-dimensional projections. The inherent limitations of planar imaging, including superimposition of bony structures and the inability to visualize deformities under weight-bearing conditions, frequently lead to misdiagnosis and suboptimal treatment planning. While weight-bearing radiographs offer some improvement, they still lack the three-dimensional (3D) spatial resolution necessary for a complete understanding of complex anatomical relationships. Past approaches focused primarily on two-dimensional analysis of weight-bearing radiographs using conventional parameters [1]. 

**Figure 1:**
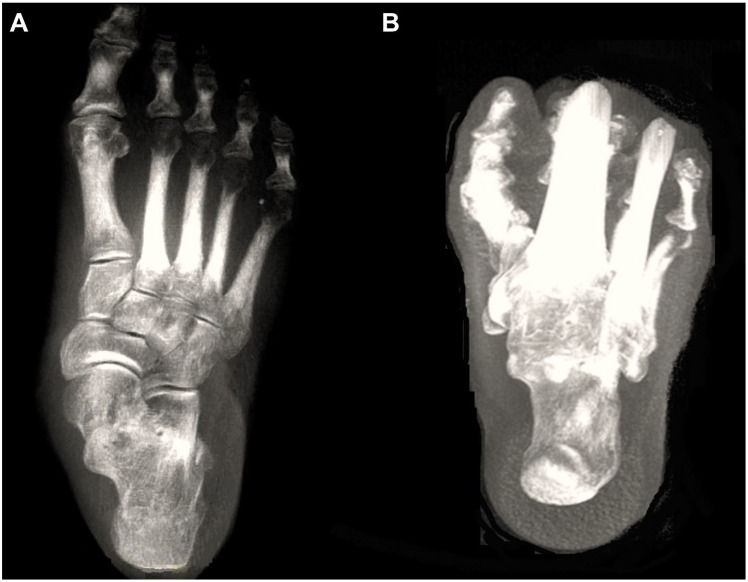
Digitally reconstructed radiographs (DRRs) demonstrating foot anteroposterior (**A**) and hindfoot alignment (**B**) views.

Recent advancements in imaging technology, specifically the development of weight-bearing computed tomography (WBCT), have been actively utilized in the field of foot and ankle surgery. In fact, with the development of digitally reconstructed radiographs (DRRs), three-dimensional (3D) processing, and the introduction of artificial intelligence (AI) technologies, WBCT may completely replace conventional radiographs in the near future (Figure 1) [2]. The advantages of WBCT include high accuracy, owing to its 3D imaging that is not affected by bone superimposition or projection angles, low radiation doses, and reduced image acquisition time due to automated processing programs [3]. However, WBCT is not without its limitations. The technology is costly and requires specialized equipment and expertise, which limits its accessibility to a select number of centers. Additionally, the increased radiation exposure, although minimized with modern systems, remains a consideration, particularly in younger or more vulnerable patient populations [2]. Patient comfort during imaging and the need for precise weight-bearing positioning can also pose challenges, especially for individuals with severe pain or mobility issues. These drawbacks highlight the need for ongoing research to enhance the affordability, accessibility, and user-friendliness of WBCT technology.

Recently, Kim et al. reviewed the use of weight-bearing CT for diseases of the ankle joint [3]. In this review, we aimed to focus on the diseases of the foot and categorized the pathologic conditions into forefoot disease (hallux valgus), midfoot disease (Lisfranc injuries and midfoot instability), and progressive collapsing foot deformity (PCFD). By critically evaluating existing studies employing WBCT, we will highlight its strengths and limitations, identify areas needing further research, and ultimately demonstrate WBCT’s role in improving the diagnosis and treatment of complex foot deformities.

## 2. Forefoot Diseases (Hallux Valgus)

Studies on WBCT in hallux valgus deformity can be classified into the following categories: application of WBCT for conventional parameters used in weight-bearing radiographs, measurement of the first metatarsal pronation, and evaluation of the first ray instability.

### 2.1. Application of Conventional Radiographic Parameters

Collan et al. were the first to apply the conventional parameters used in weight-bearing radiographs on WBCT [4]. The authors compared the hallux valgus angle (HVA) and 1–2 intermetatarsal angles (IMA) on plain radiographs with 2D and 3D angles on WBCT and found a strong correlation between the measurement methods. From this finding, the authors concluded that WBCT can be used as a primary diagnostic measure for hallux valgus because conventional data can be obtained accurately, along with the three-dimensional rotational status of the first ray. Also, while non-weight-bearing CT (NWBCT) has been traditionally used for its ability to provide detailed anatomical images without external pressure on the foot, WBCT offers unique insights into the biomechanical and functional aspects of the foot under physiological loading conditions. NWBCT provides precise anatomical details of the metatarsal bones, but WBCT offers additional information about the rotational component and first ray instability under physiological conditions [4]. With the development of technology, numerous approaches have been made to more accurately and handily identify the pathological characteristics of hallux valgus. Lalevee et al. adopted a computerized postprocessing method on the distal metatarsal articular angle (DMAA) and reported that conventional radiographs overestimate DMAA by 14 degrees [5]. They suggested that computerized pronation correction of the first metatarsal bone (M1) using WBCT would be needed to objectively evaluate the valgus status of the M1 distal articular surface. Similarly, Zhong et al. developed an innovative computer-aided design method, which showed comparable measurement reliability with conventional radiographs in terms of HVA and IMA, along with better accuracy for DMAA [6].

Moreover, research aimed at automating the measurements for hallux valgus is also being actively conducted. De Carvalho compared semi-automatic and manual measurements for radiographic parameters (HVA, IMA, and IPA (interphalangeal angle) using WBCT in hallux valgus [7]. Following manual bone segmentation by a user, the software automatically registered a mathematical model, which computed the anatomical landmarks for measurement and longitudinal axes of the selected bones. Using this method, the authors concluded that semi-automatic measurements demonstrated reproducible and comparable results to manual measurements. In a multicenter study including 128 feet from 93 patients who underwent WBCT, Day et al. compared automatically measured IMA using AI software with manually measured IMA on DRRs [8]. The authors reported that AI-based automatic measurements showed strong correlations with manual measurements, with near-perfect reproducibility. If applicable in the clinical setting, automatic measurements would enable an intuitive identification of WBCT data and prompt the development of therapeutic strategies for hallux valgus.

### 2.2. First Metatarsal Pronation

Traditionally, hallux valgus has been understood as a two-dimensional deformity, that is, a varus deviation of the first metatarsal bone (M1) and valgus orientation of the great toe [9]. As a result, classic radiographic parameters (i.e., HVA, IMA, and DMAA) that define the horizontal relationship between metatarsals and phalanges have been considered an important reference to establish a treatment strategy in hallux valgus deformity [10]. With the development in diagnostic modalities, however, a consensus has been reached that the deformity also involves a coronal component, namely, the pronation of the M1 [11]. Consequently, a three-dimensional approach should now be made to enhance the understanding of the complex triplanar deformity of hallux valgus.

**Figure 2:**
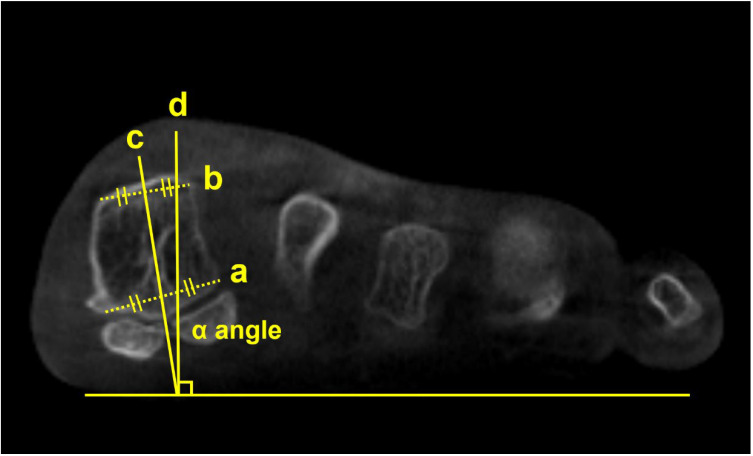
To obtain the alpha (α) angle, first, an inferior line is drawn between the lateral edge of the lateral sulcus and the medial edge of the medial sulcus (a). Subsequently, a superior line is drawn between the point of the medial and lateral corners of the first metatarsal head (b). Second, bisections of the above 2 lines are connected to a straight line perpendicular to the horizontal ground axis (c). Third, the angle is measured between the straight line (c) and the vertical line perpendicular to the ground axis (d) that is obtained from the first step.

Using semi-weight-bearing CT, Kim et al. devised the alpha angle to evaluate the pronation of the M1 [12]. It was determined on the axial view by measuring the angle between the line bisecting the M1 and the vertical line perpendicular to the horizontal ground axis (Figure 2). The authors reported that 87.3% of the patients with hallux valgus had a more pronated M1 than the control group, with a greater alpha angle. Similarly, Campbell et al. measured the M1 rotation with 3D geometrically determined angles and found that M1 pronation relative to the second metatarsal was 8.2 degrees greater in the hallux valgus group than in the control group [13]. Furthermore, Mansur et al. used WBCT to verify the round sign, an indirect sign of M1 pronation in conventional radiographs, and concluded that the round sign weakly correlated with the alpha angle measured on WBCT [14]. Overall, these findings suggest that it is challenging to fully recognize the complex 3D deformities of hallux valgus using only conventional radiographs and the incorporation of WBCT would be beneficial.

**Figure 3:**
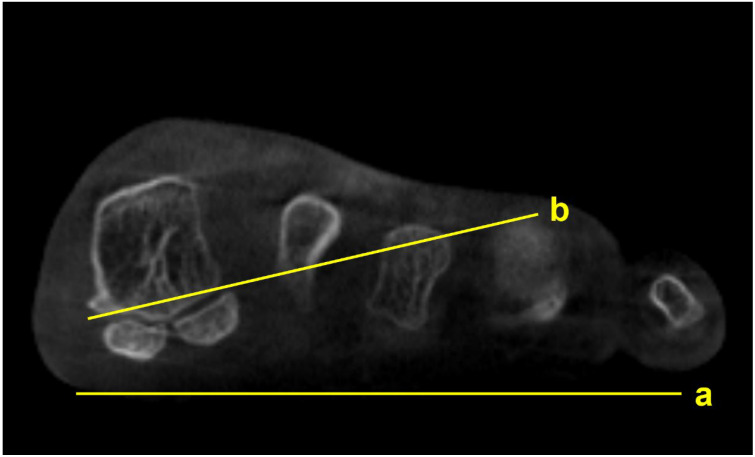
M1 pronation angle (M1PA) is the angle between the floor (a) and a line drawn from the most inferomedial border of the medial sesamoid facet to the most lateral border of the lateral sesamoid facet (b).

Several studies focused on the impact of M1 pronation after hallux surgery. Conti et al. conducted a study to determine if a postoperative decrease in M1 pronation observed in WBCT would be associated with changes in patient-reported outcomes [15]. Patients who underwent a modified Lapidus procedure for hallux valgus were divided into two groups with regards to the amount of postoperative M1 pronation change, and the Patient Reported Outcomes Measurement Information System (PROMIS) scores were compared between groups. At 2 years postoperatively, patients who had a significant decrease in M1 pronation after the modified Lapidus procedure showed greater improvement in the PROMIS physical function domain. Choi et al. utilized simulated weight-bearing CT to evaluate the association between preoperative M1 pronation and postoperative recurrence after proximal chevron osteotomy [16]. To quantify the amount of preoperative M1 pronation, the authors measured the M1 pronation angle (M1PA) (Figure 3). They reported that patients who had significant correction loss 1 year after surgery exhibited higher preoperative M1PA, with a 28.4-degree threshold. These results show the importance of recognizing preoperative M1 pronation through WBCT because the rotational component of the hallux valgus deformity would impact postoperative outcomes and recurrence rates after surgery.

### 2.3. First Ray Instability

**Figure 4:**
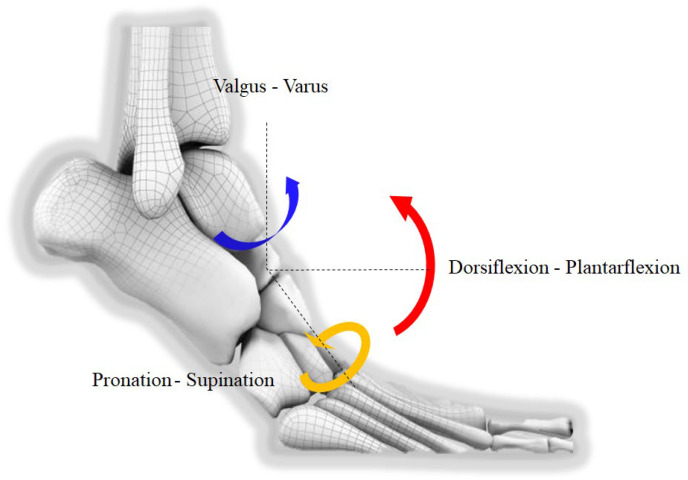
An illustration depicting 3D components of the first ray hypermobility in hallux valgus. Red arrow: Dorsiflextion-Plantarflextion, Blue arrow: Valgus-Varus, Yellow arrow: Pronation-Supination. Blue arrow: Valgus-Varus, Yellow arrow: Pronation-Supination.

Extensive research has demonstrated that hypermobility of the first ray is strongly involved in the pathogenesis of hallux valgus [17,18]. Previously, only the two-dimensional, sagittal component of the first ray mobility could be measured with conventional radiographs [19]. With WBCT, however, 3D components of the first ray hypermobility in hallux valgus could also be considered (Figure 4).

Kimura et al. used simulated WBCT and evaluated details regarding the instability of the first ray [18]. They reported that first ray instability occurs at every single joint that composes the first ray, and each joint demonstrates unique 3D motion. For instance, the hallux valgus group showed greater dorsiflexion, inversion, and adduction of the first metatarsal compared with medial cuneiform at the first tarsometatarsal (TMT) joint, along with greater eversion and abduction of the medial cuneiform than navicular bone at the medial cuneonavicular joint. Furthermore, they also evaluated displacement of the first–second intercuneiform joint in a separate study and concluded that patients with hallux valgus had greater dorsiflexion, inversion, and abduction relative to the medial cuneiform [20]. Lee et al. also investigated signs of instability of the first TMT joint on WBCT and concluded that the hallux valgus group demonstrated instability predominantly in the sagittal and axial planes [21]. Based on the results of the aforementioned studies, WBCT could be regarded as a valuable tool to accurately capture the 3D instability of the first ray in hallux valgus and to determine the necessity of surgical intervention.

### 2.4. Summary

In conclusion, WBCT offers significant advantages over conventional radiography in the diagnosis and management of hallux valgus (Table 1). Its ability to provide detailed 3D visualization, coupled with automated measurement techniques and AI integration, allows for a more precise assessment of the deformity’s complexity, including hallux valgus angle, intermetatarsal angle, and metatarsal pronation. This improved diagnostic accuracy facilitates better surgical planning and potentially leads to improved patient outcomes. Further research is needed to fully standardize WBCT measurement protocols and optimize the clinical application of AI-assisted analysis.

## 3. Midfoot Disease

### 3.1. Lisfranc Injuries

Lisfranc injury indicates injuries on the tarsometatarsal joint of the foot resulting from low-energy-induced ligamentous injury to high-energy-induced fracture or dislocation [22]. Among the injury spectrum, low-energy-induced subtle Lisfranc injuries are often misdiagnosed initially because these are presented without substantial radiographic abnormality on the weight-bearing radiographs [23]. In fact, owing to the biomechanical importance of the Lisfranc joint as a keystone in the foot arch, even a subtle injury should be diagnosed precisely and managed properly [24]. Although conventional CT or MRI can be accompanied to overcome the relatively low sensitivity, the unloaded condition may not fully demonstrate the physiologic property of the midfoot [25,26]. Sripanich Y. et al. reported that subtle Lisfranc injuries, which are often missed on NWBCT, can be diagnosed with greater sensitivity using WBCT due to its ability to reveal ligamentous instability and joint widening under stress [27].

Recently, multiple studies have reported the use of WBCT in Lisfranc injuries. Sripanich et al. conducted an experiment on 24 intact cadaveric feet to investigate the amount of Lisfranc ligamentous complex (LLC) joint widening after injury under different loading conditions [28]. They found that Lisfranc joint widening greater than 1.5 mm under partial weight-bearing conditions on WBCT could be regarded as a complete Lisfranc injury. In fact, additional adjacent ligament injury was needed for Lisfranc joint widening to be greater than 2 mm, which is a well-known diagnostic cutoff value on conventional radiographs. This finding indicates that isolated Lisfranc ligament injury could be overlooked in conventional radiographs if a 2 mm widening was used as a radiographic threshold.

To enhance the diagnostic accuracy, Campbell et al. proposed an augmented stress weight-bearing CT to detect subtle, dynamically unstable Lisfranc injuries [29]. With weight bearing on both feet facing forward, the patient was asked to raise both heels from the scanner platform. This plantarflexion force on the midfoot provides augmented stress on the midfoot, which improves the sensitivity in identifying subtle Lisfranc injuries.

**Figure 5:**
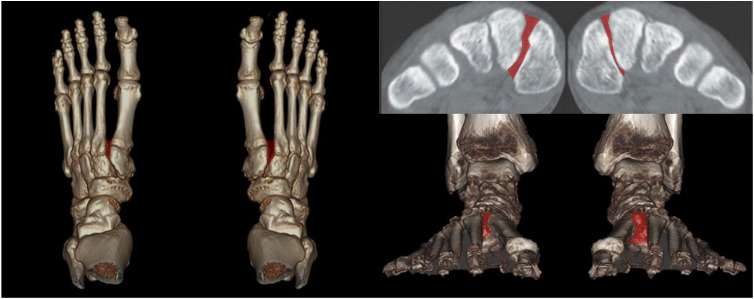
3D volumetric measurement of the Lisfranc joint.

Instead of conventional axial measurement of the Lisfranc joint, some novel WBCT parameters have been developed and introduced for use in the clinical setting. Sripanich evaluated 96 cadaveric specimens and designed a WBCT protocol to enhance the reliability of Lisfranc joint measurements [29]. They found that measuring the distance between the medial cuneiform and second metatarsal with coronal WBCT imaging would be a reproducible way to localize the interosseous Lisfranc ligament injury. Similarly, Bhimani et al. evaluated the Lisfranc joint complex using one-dimensional (1D), two-dimensional (2D), and 3D measurements on WBCT scans among operatively confirmed Lisfranc instability (Figure 5) [30]. They concluded that coronal 3D volumetric measurement had higher sensitivity and specificity than 2D and 1D measurements because the second metatarsal tends to displace both laterally and superiorly in Lisfranc injury. Despite its inherent limitation in being actively used in acute conditions, WBCT may enhance diagnostic accuracy for suspicious Lisfranc injuries with uncertain conventional radiograph findings.

### 3.2. Midfoot Osteoarthritis

**Figure 6:**
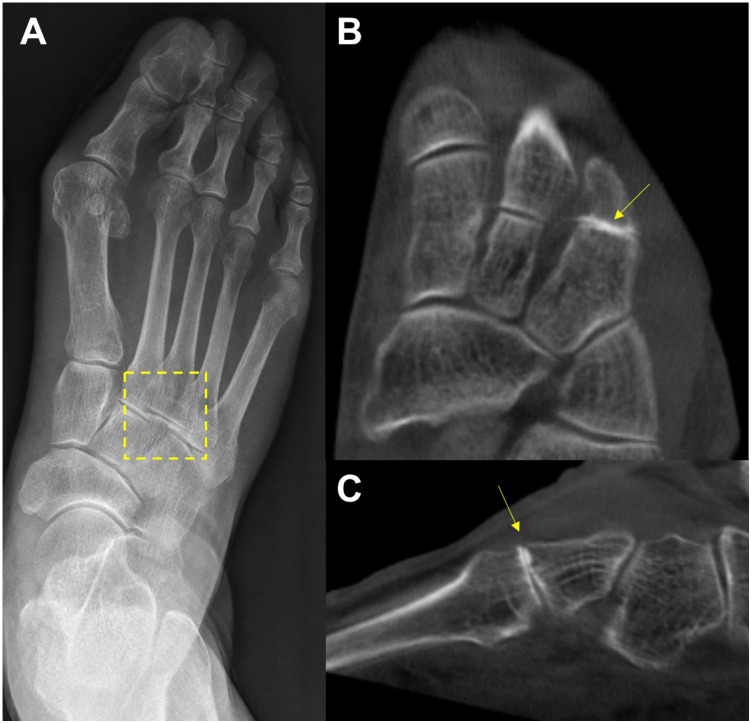
In the plain radiograph, osteoarthritis of the third tarsometatarsal (TMT) joint is not clearly visible (**A**, See box area); however, weight-bearing computed tomography (WBCT) reveals distinctive joint space narrowing and sclerotic changes (**B**,**C**; See arrow).

In the midfoot, precise identification of associated articulations and osseous borders in conventional weight-bearing radiographs may not be handy because they can be affected by overlapping adjacent bones when viewed two-dimensionally [31] (Figure 6). For this reason, WBCT is an alternative, as it enables clear joint space visualization and enhanced bony landmark identification under physiological weight-bearing conditions. Steadman et al. compared weight-bearing radiographs and WBCT with regard to diagnostic accuracy in midfoot osteoarthritis [32]. They found that weight-bearing radiography demonstrated 61.5 to 72.5% sensitivity and 87.9 to 96.1% specificity in identifying midfoot osteoarthritis. It also showed less accurate localization of degenerative changes and a greater tendency to underestimate disease severity compared to WBCT. These findings indicate that WBCT would be a better diagnostic option in midfoot osteoarthritis as it provides an earlier and more reliable diagnosis.

With the support of such high accuracies achieved by WBCT, Kim et al. aimed to re-establish the prevalence of midfoot arthritis [33]. Analyzing 606 patients who underwent WBCT for foot and ankle problems, the authors detected that 57.9% of the patients had midfoot arthritis, which is higher than previous studies that were based on surveys, physical examination, and conventional radiographs. The authors also confirmed the related factors associated with medical history and comorbid foot deformities for midfoot arthritis, which was previously determined based on conventional radiographs [34]. They concluded that older age, right sidedness, increased body mass index (BMI), PCFD, and lateral toe deformities contribute to higher possibilities of midfoot arthritis, based on WBCT. Given the significant differences compared to previous results, we believe that WBCT should be more actively used in the clinical assessment of patients presenting with dorsal foot pain.

### 3.3. Summary

The findings presented here clearly demonstrate the superiority of WBCT in identifying subtle Lisfranc injuries and characterizing midfoot osteoarthritis compared to conventional methods (Table 2). The use of stress WBCT and novel measurement techniques, combined with the increased spatial resolution of WBCT, substantially improves diagnostic accuracy and allows for the early detection and appropriate management of these conditions. Further studies focusing on the standardization of WBCT protocols and the development of predictive models based on WBCT data are warranted.

## 4. Progressive Collapsing Foot Deformity

In 2020, Myerson et al. proposed the term “Progressive Collapsing Foot Deformity (PCFD)” and a new classification system to summarize the adult-acquired flatfoot deformity [35]. The new system includes the terms “progressive” and collapsing” to give a better idea of the worsening and evolving nature of the complexity of the 3D deformity. It covers varying degrees of hindfoot valgus (Class A), mid/forefoot abduction (Class B), medial column instability (Class C), peritalar subluxation (Class D), and ankle instability (Class E). Given these points, WBCT could be a highly effective tool for interpreting and classifying a complex 3D deformity within PCFD.

### 4.1. Hindfoot Valgus Deformity (Class A)

As in plain radiographs, the hindfoot alignment angle (HAA) and hindfoot moment arm (HMA) are commonly used measurements in WBCT as indicators for assessing hindfoot valgus in PCFD [36]. de Cesar Netto et al. reported that clinical examination of the HAA tends to underestimate the extent of hindfoot valgus and suggested that WBCT measurements would be more reliable and repeatable [37]. These parameters, however, are limited in that they cannot account for the fact that forefoot deformity can act as a contributor to hindfoot deformity [38].

**Figure 7:**
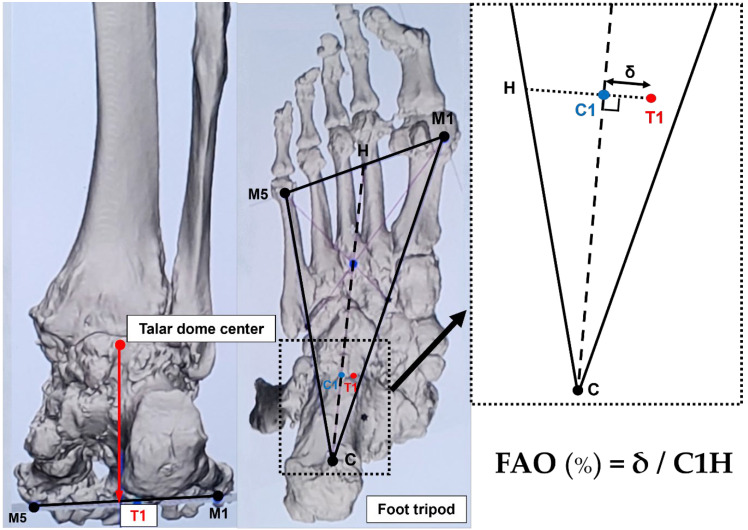
Foot–ankle offset (FAO). First, the most plantar aspect of the M1, the most plantar aspect of the M5, and the most plantar aspect of the calcaneal tuberosity (C) are marked to create the foot tripod. Then, the talar dome center (T1) is established on the foot tripod. The FAO represents the percentage of ankle deviation from the tripod’s center (C1) (Figure 2).

As an alternative, FAO is considered a validated measurement demonstrating the relationship between the center of the ankle joint and the center of the tripod of the foot, which can be calculated semiautomatically using WBCT (Figure 7) [39]. Because it simultaneously reflects the alignment of the hindfoot, midfoot, and forefoot, FAO is being widely used to measure overall 3D foot deformities. Lintz et al. reported that FAO values greater than 4.6% have a specificity of 100% and a sensitivity of 89.2% for the diagnosis of PCFD [40]. Day et al. compared the FAO values before and after surgery in the feet of 20 PCFD patients, showing a significant difference from 9.8% before surgery to 1.3% after surgery [41]. Consequently, FAO is considered a critical measurement modality in evaluating the overall PCFD before surgery and predicting the extent of correction after surgery.

### 4.2. Midfoot/Forefoot Abduction (Class B)

**Figure 8:**
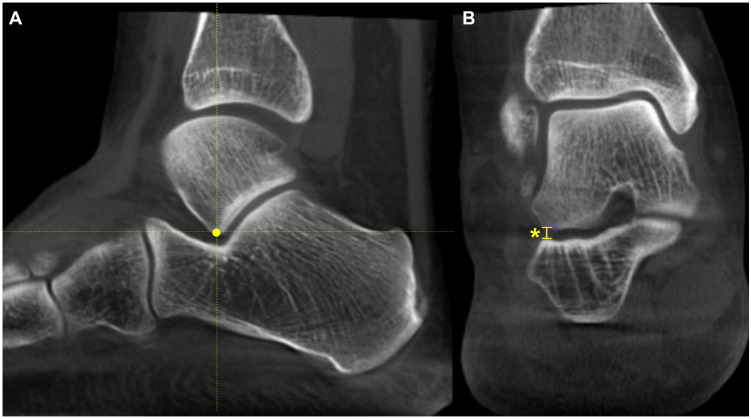
Talocalcaneal distance. (**A**) Using the sagittal view for reference, the most inferior point of the lateral process is identified. (**B**) At this point, the narrowest distance between the inferior border and the calcaneal floor is measured in the selected coronal slice (Asterisk).

The talonavicular coverage angle is also used in WBCT as an indicator to assess midfoot abduction [36]. In fact, a new classification system for PCFD includes sinus tarsi impingement as one of the findings of midfoot abduction. Sinus tarsi impingement, which commonly causes lateral hindfoot pain, is caused by bony contact between the talus and calcaneus and should be addressed when establishing a therapeutic strategy in PCFD [42]. Because of superimposition effects, however, sinus tarsi impingement is difficult to identify using conventional radiographs. Instead, WBCT allows for the identification of bony impingement in a physiological standing position. Kim et al. devised a novel method to measure the talocalcaneal distance, which features realignment of the coronal and sagittal planes to directly trace the inferior border of the lateral process of the talus [43] (Figure 8). The inferior border of the lateral process of the talus is chosen to reconstruct the coronal reference plane because it is a constant anatomical landmark to obtain minimal talocalcaneal distance. Using this method, the authors investigated the correlation of talocalcaneal distance narrowing with common radiographic parameters on standard weight-bearing radiographs. They observed that talocalcaneal narrowing correlated most with talonavicular coverage, with a cutoff value of 41.2 degrees. Andres et al. conducted a comparative study to identify whether there is an association between WBCT-based measurements and MRI findings [44]. They found that MRI findings overestimate the presence of bony sinus tarsi impingement in approximately 42% of the included population and concluded that WBCT would be a better diagnostic option to detect bony impingement in PCFD.

**Figure 9:**
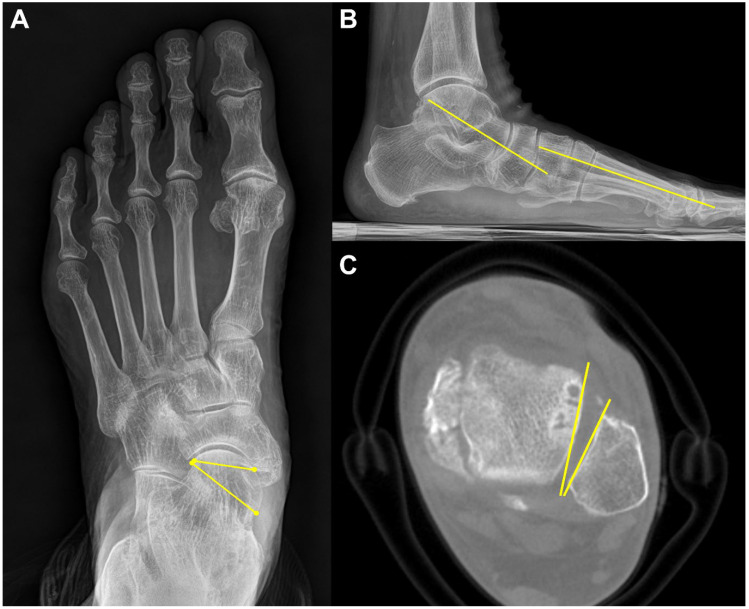
A 71-year-old male PCFD patient with 35-degree talonavicular coverage angle (**A**) and 20-degree lateral Meary’s angle (**B**). The talus is internally rotated with regard to lateral malleolus on the weight-bearing CT axial image (**C**).

Lastly, Kim et al. suggested that talar malrotation in the axial plane should be considered an underlying feature of abduction deformity in PCFD [45]. They detected that the talus was significantly more internally rotated in reference to the lateral malleolus, the ankle transmalleolar axis, and the lateral malleolus in PCFD patients compared to controls (Figure 9). Moreover, the severe abduction group (talonavicular coverage angle (TNC) > 40 degrees) showed more internal rotation compared with the moderated abduction group (TNC 20 to 40 degrees). With the use of WBCT, surgeons can take axial components of PCFD into consideration at the time of reconstructive surgery in PCFD.

### 4.3. Medial Column Instability (Class C)

**Figure 10:**
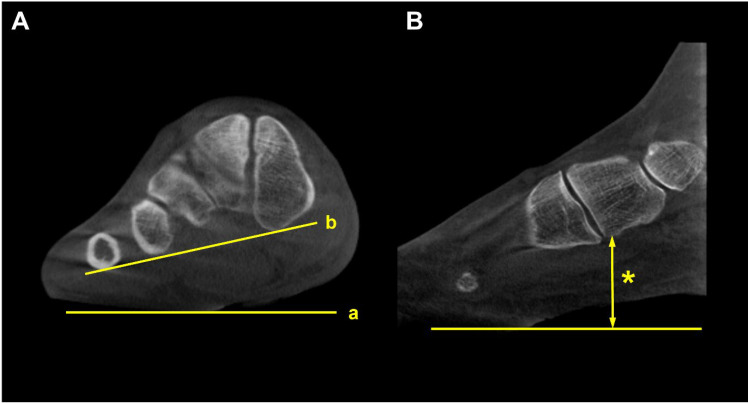
Forefoot arch angle (FFA) and medial cuneiform-to-floor distance (MCFD). (**A**) FFA is defined as an angle between the floor (a) and the line connecting the most plantar aspect of the medial cuneiform and 5th metatarsal (b). (**B**) MCFD is measured from the most plantar aspect of the medial cuneiform to the floor (asterisk).

Forefoot arch angle (FAA) and medial cuneiform-to-floor distance (MCFD) are the measurements used to evaluate medial column instability in PCFD [36]. FFA is determined in the coronal plane by establishing a line from the most plantar aspect of the medial cuneiform to the fifth metatarsal. The angle between this line and the ground is defined as FAA. MCFD is measured in the sagittal plane from the most plantar aspect of the medial cuneiform to the ground plate (Figure 10). de Cessar Netto et al. reported that both FAA and MCFD reflect medial column instability with almost perfect reliability [36]. In non-weight-bearing and weight-bearing CT scans, the MCFD measured 29 mm and 18 mm, respectively, and the FFA measured 13 degrees and 3 degrees, respectively, indicating medial column instability with differences observed under weight-bearing conditions.

### 4.4. Peritalar Subluxation (Class D)

**Figure 11:**
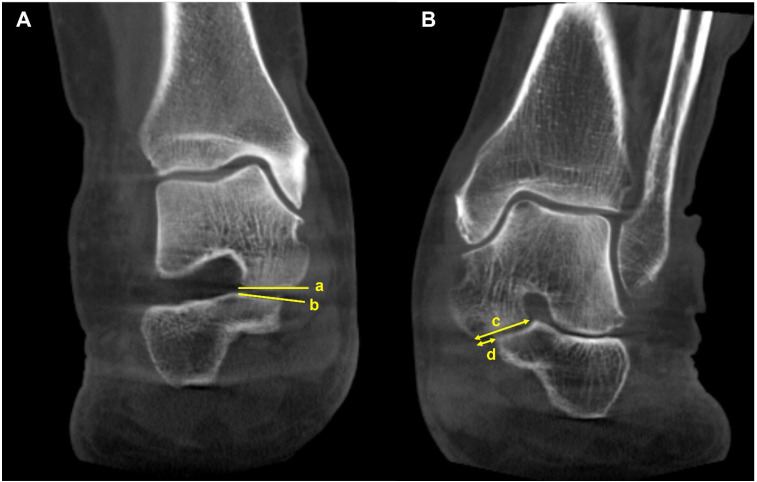
Middle facet subluxation. (**A**) Measurement of the incongruence angle of the middle facet of the subtalar joint on a coronal-plane weight-bearing CT image, which is an angle between both articular surfaces (a and b). (**B**) Measurement of the percentage of “uncoverage” of the middle facet of the subtalar joint on a coronal-plane weight-bearing CT image. (c) = the width of the talar middle facet, and (d) = the linear measurement of the middle facet uncoverage. The percentage of uncoverage of the middle facet of the subtalar joint = (d/c).

Peritalar subluxation is known to be a key pathological index in PCFD. It defines the complex 3D distortion that occurs in PCFD and is characterized by subluxation of the hindfoot through the triple joint complex [46]. The percentage of middle facet subluxation (MFS) and incongruence angle are considered validated markers for peritalar subluxation measured in WBCT [46]. Assessed at the midpoint of the subtalar joint middle facet in the sagittal plane, uncoverage of the middle facet on a coronal plane image is measured. Then, the percentage of MFS is measured by dividing the uncoverage of the middle facet by the width of the talar middle facet. The incongruence angle is the angle between both articular surfaces at the midpoint of the middle facet of the subtalar joint (Figure 11). De Cesar Netto et al. reported that an incongruence angle of >8.4° and an MFS percentage of >17.9° were found to be highly diagnostic for symptomatic stage II adult-acquired foot deformity [46]. The authors also compared the amount of subluxation of the middle and posterior facets of the subtalar joint to identify the superior marker to detect early peritalar subluxation [47]. They reported significantly pronounced subluxation of the middle facet than that of the posterior facet by an average of 17.7%, which implies that MFS may present an earlier and more significant sign of progressive peritalar subluxation.

**Figure 12:**
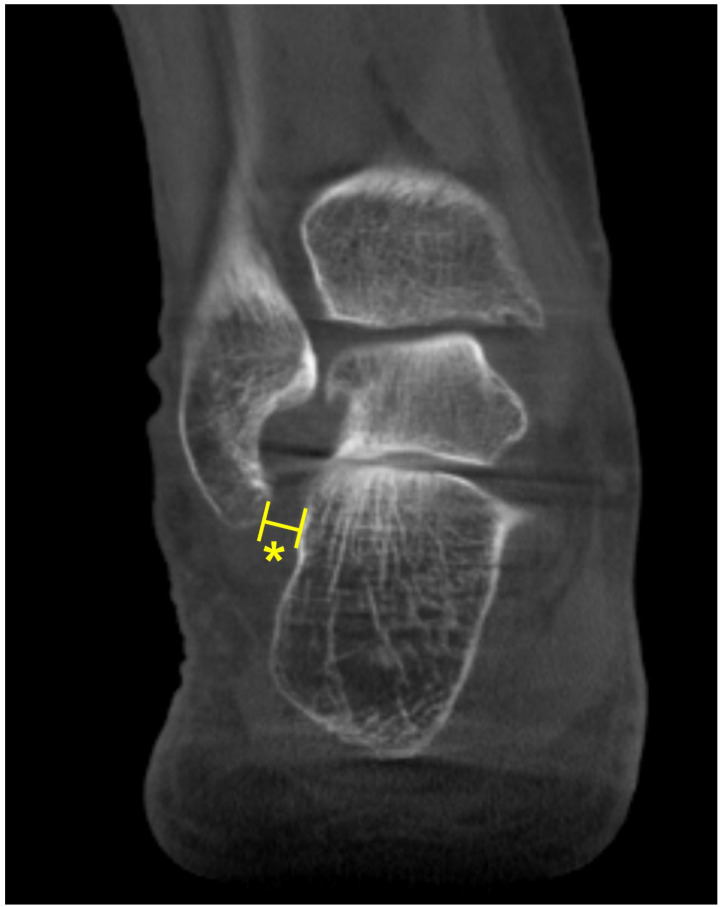
Calcaneofibular distance is the shortest distance from the fibula to the lateral margin of the posterior facet of the calcaneus in the coronal image (asterisk).

Subfibular impingement is also one of the findings characterizing peritalar subluxation in PCFD. To address this, Jeng et al. devised a calcaneofibular distance measured on the WBCT coronal view (Figure 12) [48]. The calcaneofibular distance is defined as the closest distance between the lateral aspect of the posterior facet of the calcaneus, which is the most reproducible point to measure from the calcaneus lateral wall and the fibula. Using this method, Kim et al. observed that subfibular impingement detected on the WBCT correlated best with HMA in the weight-bearing radiograph [43]. They also added that HMA cutoff values of 25.4 mm and 38.1 mm would be useful for ruling out and diagnosing calcaneofibular impingement, respectively.

### 4.5. Valgus Tilting (Class E)

Myerson et al. described ankle valgus deformity as a continuum of posterior tibial tendon dysfunction (PTTD) and classified this condition as the final stage of PTTD, known as stage IV [49]. However, ankle valgus is caused by the rupture of the deltoid ligament rather than being a continuation of PTTD and can be considered an independent feature of PCFD [50]. As a result, in a new classification, ankle valgus is described as Class E, one of the five independent features of PCFD.

Mansur et al. conducted a study to see if traditional hallmarks of peritalar subluxation could be adopted in patients with class E deformity in PCFD [51]. They detected a paradoxical reduction of peritalar subluxation despite underlying peritalar ligamentous incompetence. The authors interpreted that the deformity fulcrum would be changed proximally in class E. They recommended using FAO as an imaging parameter in class E and concluded that FAO greater than 12.14% is a strong predictor of ankle valgus deformity in PCFD.

### 4.6. Transverse Arch Collapse

**Figure 13:**
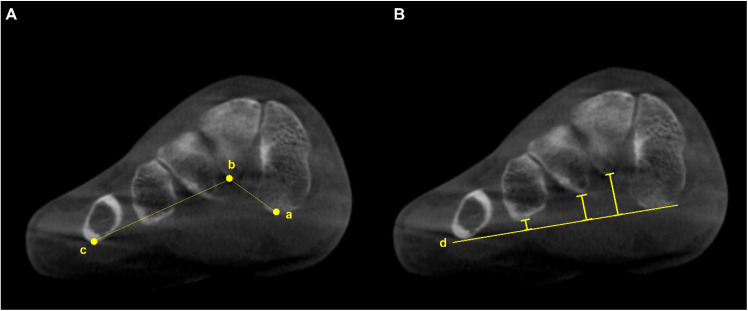
(**A**) Transverse arch plantar (TAP) angle represented by the angle between the most plantar parts of the 1st (a), 2nd (b), and 5th (c) TMT joints. (**B**) Location of collapse along the transverse arch is determined by comparing the distance between the line connecting the most inferior aspect of the medial cuneiform and the 5th metatarsal bone (d) and specific components of the transverse arch.

In addition to the collapse of the medial longitudinal arch, Schmidt et al. focused on the transverse arch in PCFD [52]. They defined the transverse arch plantar (TAP) angle to evaluate the angle formed between the first, second, and fifth metatarsals in the coronal plane, which was significantly higher in PCFD. Furthermore, the authors measured the distance between the bones composing the transverse arch and a line connecting the most inferior aspect of the medial cuneiform and the fifth metatarsal. The location of collapse along the transverse arch was most prominent at the second metatarsal and medial cuneiform (Figure 13). In this way, WBCT proves to be useful with regard to converting multiple points on the coronal plane into lines within a single plane and assessing the angles formed by these lines to determine the amount of transverse arch collapse. Additionally, it allows for the identification of the location of arch collapse in specific planes that may be obscured by bone superimposition in conventional radiographs.

### 4.7. Distance Mapping in PCFD

Distance mapping is a recently validated technology to quantify and compare joint surface interaction by postprocessing the WBCT data [53]. It visualizes the joint surface distance distribution through a color-coded map and enables the identification of relative positions between joint surfaces three-dimensionally. Since PCFD is characterized by abnormal subluxation and impingement of bones around multiple joints within the foot, distance mapping is being widely used to objectively quantify the amount of deformity [53]. Furthermore, the concept of coverage mapping has been suggested by Dibbern et al. to better highlight areas of proper joint interaction, joint subluxation, and impingement [53]. This method distinguishes articular coverage, bony impingement, periarticular interaction, and the shadow of the talus using distinct colors, which would enhance the interpretability and clinical utility of distance mapping in PCFD. Using coverage mapping, the authors reported decreased articular coverage in articular regions and increased impingement in nonarticular regions in patients with PCFD [54]. In detail, they found significantly increased uncoverage in the middle facet, not in the anterior or posterior facets, and significantly increased sinus tarsi coverage along with impingement. In a subsequent study, the authors detected significantly decreased articular coverage of the anterior aspect of the ankle gutters and talar dome in PCFD patients, which is consistent with early plantarflexion of the talus within the ankle mortise. Through the advanced techniques processing WBCT data, clinicians are now able to achieve a more objective understanding of the complex three-dimensional deformities in PCFD.

### 4.8. Summary

In PCFD, the use of WBCT-specific measurements like FAO, coupled with innovative techniques such as distance mapping, allows for a more precise characterization of the deformity’s severity and its various components (Table 3). This improved understanding facilitates more accurate surgical planning and the potential for more personalized treatment strategies tailored to the specific aspects of the individual patient’s deformity.

## 5. Conclusions

WBCT is poised to become the gold standard in foot and ankle imaging due to its unparalleled diagnostic capabilities. Its integration into clinical practice complements the detailed anatomical imaging provided by NWBCT, offering a more holistic view of complex pathologies. However, overcoming barriers such as cost and accessibility is crucial for its broader adoption. Technological advancements in conventional imaging methods, including the incorporation of AI and three-dimensional postprocessing algorithms, could bridge the gap in settings where WBCT is unavailable, although they cannot yet fully replicate its precision [55,56]. Future directions should focus on making WBCT more accessible and cost-effective, potentially through the development of portable systems and streamlined protocols. Simultaneously, enhancing WBCT’s integration with AI could automate diagnostics and improve accuracy. Expanding its applications in surgical planning, postoperative evaluations, and personalized treatment strategies has the potential to revolutionize patient care.

In conclusion, while WBCT offers the most comprehensive diagnostic insights for complex deformities, complementary advancements in alternative imaging methods could serve as valuable tools to address accessibility challenges. Ensuring the widespread availability of WBCT and refining supplementary technologies will ultimately lead to improved diagnostic accuracy and better clinical outcomes for a broader patient population.

## Figures and Tables

**Table 1:** Advantages of WBCT measurement parameters in hallux valgus.

Measurement Parameter	Advantages
HVA	More precise measurement in 3D
IMA	More precise and less affected by superimposition
DMAA	More accuracy in 3D (conventional radiographs overestimate DMAA)
M1PA	Captures a key component of the deformity not visible on 2D images

HVA, hallux valgus angle; IMA, intermetatarsal angle; DMAA, distal metatarsal articular angle; M1PA, first metatarsal pronation angle.

**Table 2:** Advantages of WBCT in midfoot diseases.

Midfoot Diseases	Measurement Parameter	Advantages
Lisfranc injury	Lisfranc joint widening, 3D displacement	Detection of subtle injuries under weight-bearing conditions, assessment of ligamentous injury
Midfoot osteoarthritis	Joint space width at various joints	Improved visualization of joint space, assessment of articular cartilage, accurate measurement of joint space narrowing

**Table 3:** Advantages of WBCT measurement parameters in PCFD.

PCFD Class	Measurement Parameter	Advantages
A (Hindfoot valgus)	HAA, HMA, FAO	assessment of 3D deformity, precise measurement of hindfoot alignment
B (Midfoot/forefoot abduction)	TNC, talocalcaneal distance	assessment of midfoot abduction, detection of sinus tarsi impingement
C (Medial column instability)	MCFD, FAA	assessment of medial column collapse and instability
D (Peritalar subluxation)	MFS, incongruence angle, calcaneofibular distance	assessment of peritalar subluxation and subfibular impingement, quantification of middle facet subluxation
E (Ankle valgus)	FAO (in advanced cases)	assessment of ankle valgus and its contribution to overall deformity

PCFD, progressive collapsing food deformity; HAA, hindfoot alignment angle; HMA, hindfoot moment arm; FAO, foot–ankle offset; TNC, talonavicular coverage angle; MCFD, medial cuneiform-to-floor distance; FAA, forefoot arch angle; MFS, percentage of middle facet subluxation.
